# The Neuro-Inflammatory-Vascular Circuit: Evidence for a Sex-Dependent Interrelation?

**DOI:** 10.3389/fnins.2020.614345

**Published:** 2020-12-09

**Authors:** Catherine Gebhard, Susan Bengs, Achi Haider, Michael Fiechter

**Affiliations:** ^1^Department of Nuclear Medicine, University Hospital Zurich, Zurich, Switzerland; ^2^Center for Molecular Cardiology, University of Zurich, Zurich, Switzerland; ^3^Swiss Paraplegic Center, Nottwil, Switzerland

**Keywords:** cardiovascular, autonomous nervous system, inflammation, sex, limbic system

## Abstract

Cardiovascular disease (CVD) is the leading cause of death worldwide with mortality rates in women currently exceeding those in men. To date, evidence is widely lacking for unique female determinants of CVD. However, strong associations with psychological stress, obesity or elevated inflammatory biomarkers with adverse cardiovascular outcomes in women have been identified in various studies. Interestingly, amygdalar metabolic activity, a central neural structure involved in emotional stress processing, has proven to be an independent predictor of major adverse cardiovascular events (MACE). Moreover, upregulated amygdalar metabolism was directly linked to myocardial injury in women, but not in men. This newly suggested sex-dependent brain-heart interrelation was further supported by the discovery that bone marrow activity, a surrogate parameter of inflammation, represents a potential bridging link between amygdalar activity and cardiovascular pathology by fueling inflammatory processes that promote atherosclerotic disease. Such malignant cascade of events might account, at least in part, for the excess female mortality seen in women with coronary artery disease and calls for sex-specific research toward pharmacologic or behavioral modulators to improve cardiovascular outcomes, particularly in women. This mini review summarizes recent advances in cardiovascular sex-specific medicine, thereby focusing on the interplay between the limbic system, autonomic regulation and inflammatory biomarkers, which may help to tailor CVD management toward the female cardiovascular phenotype.

## Introduction

While the term “sex” is used within the context of biological differences that constitute the female and male characteristics of anatomy and physiology, the term “gender” typically refers to the social and cultural differences between women and men (Wizemann and Pardue, 2001). Indeed, a plethora of studies have consistently reported sex and gender differences in various pathologies (Miller, 2014). As such, initial efforts have been made to personalize medicine toward sex- and gender-specific differences, yet, much remains to be elucidated to identify reliable determinants that can be incorporated into current risk stratification and clinical guidelines.

**FIGURE 1:**
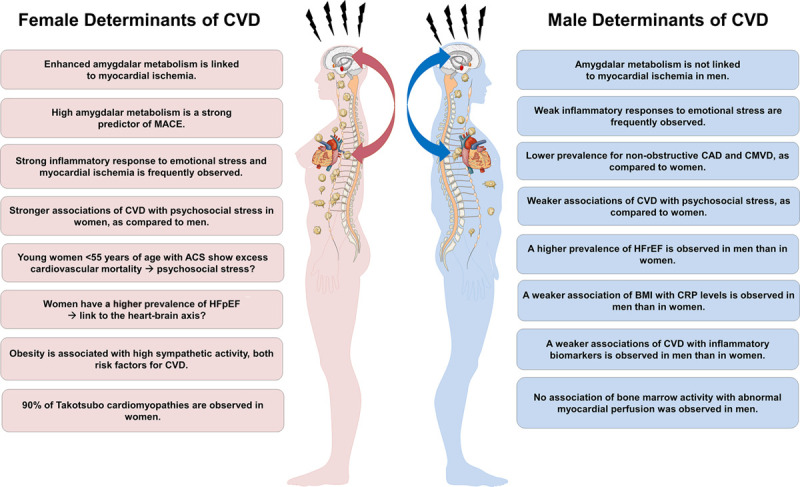
Potential involvement of the heart-brain axis in sex-dependent risk factors and presentations of cardiovascular disease (CVD). BMI, body mass index; CAD, coronary artery disease; CMVD, coronary microvascular dysfunction; CRP, C-reactive protein; MACE, major adverse cardiac events; ACS, acute coronary syndromes; HFpEF, heart failure with preserved ejection fraction; HFrEF, heart failure with reduced ejection fraction.

Sex and gender differences play a major role for the diagnosis and management of cardiovascular disease (CVD). Indeed, CVD mortality rates in women in Europe have surpassed those in men (Shaw et al., 2009; Wilmot et al., 2015; Townsend et al., 2016). Women develop CVD with similar frequency as men do, but on average 10 years later in life (Garcia et al., 2016). Once women experience an acute coronary syndrome (ACS), however, their prognosis is worse than that of men, even when adjusting for age differences (Haider et al., 2020). Notably, mortality rates of ACS in women below 55 years of age are currently increasing, while fatality decreased in age-matched men (Gabet et al., 2017; Arora et al., 2019). Although mechanisms behind the increasing cardiovascular vulnerability of women are not yet understood, there is a growing body of evidence for sex and gender differences in cardiovascular risk factors, treatment efficacy, and cardiovascular outcomes (Pagidipati and Peterson, 2016; Vaccarino, 2019). As such, epicardial coronary arteries are smaller in women than in men, independent of adjustment for body mass index, left ventricular dimensions and age (Hiteshi et al., 2014). Further, parameters of myocardial blood flow (MBF), as determined by positron emission tomography, such as baseline and hyperemic MBF are higher in women than in men, thus leading to a comparable global coronary flow reserve in women and men (Murthy et al., 2014; Haider et al., 2019a). The smaller diameter of epicardial coronary arteries and higher baseline MBF in women is proposed to result in higher endothelial shear stress in female coronaries (Patel et al., 2016). As low endothelial shear stress predisposes for regional lipid accumulation, pathologic plaque remodeling, and atherosclerotic plaque instability (Koskinas et al., 2013), it is suggested that the condition of increased shear stress in women’s coronary arteries could explain sex disparities in coronary artery disease (CAD) susceptibility (Kerkhof and Miller, 2018). The traditional focus of cardiovascular research on the identification and treatment of epicardial obstructive CAD, a condition more frequently observed in males than in females, and the detection of the “vulnerable” atherosclerotic plaque as the primary cause of ischemic injury might further explain the decreasing mortality rates in men, but not in women (Arbab-Zadeh and Fuster, 2015; Haider et al., 2020). Women tend to display a more diffuse and non-obstructive coronary artery disease pattern with distinct plaque characteristics such as lower overall plaque burden, coronary calcium content, and less necrosis in the core of the plaque (Lansky et al., 2012). In the absence of obstructive CAD, coronary microvascular dysfunction (CMVD) has been associated with an increased frequency of hypertrophic cardiomyopathy and thus, the development of adverse cardiac events such as heart failure, arrythmias, and sudden cardiac death (Crea et al., 2014). Of note, CMVD is more frequently observed in women than in men (Dean et al., 2015). Further, CMVD is associated with sympathetic hyperactivity, which in turn contributes to a reduced event-free survival in women, but not in men (Gebhard et al., 2019; Maredziak et al., 2020). In addition, elevated baseline levels of inflammatory biomarkers and a more pronounced inflammatory response to emotional stress-induced myocardial ischemia have both been observed primarily in women (Sullivan et al., 2018). An overview of the potential involvement of the heart-brain axis in sex-dependent risk factors and presentations of CVD is provided in Figure 1.

In this mini review, we aim to highlight neurohumoral sex differences that have been recently linked to CVD. As such, this review focuses on the interplay between the limbic system, autonomic dysregulation and inflammation within the context of CVD.

## Amygdalar Activity and the Female Heart

The female propensity toward worse outcomes has prompted a call to reconsider current and traditional risk-stratification methods in cardiovascular medicine (Bairey Merz et al., 2017). In particular, concerns have been raised about the excess female vulnerability toward non-traditional risk factors such as unrecognized psychosocial factors, sex-specific neural, and vascular stress responses. Recent investigations demonstrate a higher burden of emotional stress following an ACS and a predominant occurrence of Takotsubo cardiomyopathy in women (Vaccarino and Bremner, 2017; Vaccarino et al., 2018) suggesting a sex-specific neuro-cardiac link. Takotsubo cardiomyopathy leads to transient left ventricular dysfunction as a result from severe psychological stress. Moreover, impaired connectivity of brain structures involved in the control of the autonomous nervous system has been detected in patients with Takotsubo cardiomyopathy (Templin et al., 2019). Although Takotsubo cardiomyopathy is responsible for only 3% of ACS cases, the prevalence in postmenopausal women is twice as high (Ghadri et al., 2018). An important role in processing higher brain functions such as cognition and emotion is attributed to the brain’s ventral-attention or salience network (Sevinc et al., 2017). It is primarily composed of the anterior insula and the dorsal anterior cingulate cortex. Independent component analysis using functional magnet resonance imaging identified further associated structures including the amygdala, a central structure of the limbic system (Wang et al., 2010). Of note, efferent projections of the amygdala to the brain stem were found to be engaged in the sympathetic stress response (LeDoux et al., 1988). A recent study concluded that amygdalar metabolic activity is an independent risk predictor for major adverse (MACE) over and above conventional cardiovascular risk factors (Tawakol et al., 2017). As a potential mechanism, an increased inflammatory state as witnessed by upregulated bone marrow activity and arterial inflammation has been suggested. This hypothesis is further supported by the observation that increased inflammation enhanced cardiac remodeling via upregulation of interleukin-18 and myocardial β-adrenergic receptor hyper-activation in an experimental model (Xiao et al., 2018).

A recent study focusing on myocardial function, perfusion, and metabolic amygdalar activity revealed an association between elevated amygdalar activity and impaired myocardial perfusion/function. Notably, these associations were observed in women, but not in men (Fiechter et al., 2019a). Further, resting amygdalar activity was not affected by high coronary artery calcium scoring, suggesting that the extend of myocardial injury, rather than atherosclerotic plaque burden, is mainly responsible for an enhanced neural stress response in the female population. Together with the findings provided by Tawakol et al. (2017), this investigation strengthens the hypothesis that a disproportionate burden of emotional stress might contribute to worse cardiovascular outcomes in women with ACS.

Finally, Mehta et al. (2019) proposed a malignant cascade that interconnects the limbic system via the hypothalamus to neurohormonal systems (cortisol and norepinephrine), which in turn upregulates heart rate and blood pressure. This may ultimately lead to the activation of inflammatory processes, endothelial dysfunction, and atherosclerosis, thereby increasing the risk of ACS, heart failure, and sudden cardiac death.

## Altered Autonomous Nervous System Activity and Upregulated Inflammatory State: The Missing Piece of the Puzzle?

An enhanced sympathetic tone has recurrently been observed in women with CMVD, Takotsubo cardiomyopathy (Di Monaco et al., 2010; Templin et al., 2015), and ACS (Ubrich et al., 2017), and was associated with adverse cardiovascular outcomes (Hogarth et al., 2009). Similarly, worse prognoses have been documented in women with heart failure and myocardial infarction and were found to be related to upregulated cardiac sympathetic activity (La Rovere et al., 1998; Mitoff et al., 2011). Of note, sympathetic activity remained elevated until 9 month after an ACS in women, adding to an unfavorable prognosis (Hogarth et al., 2009). Cardiac autonomic dysregulation in women with major depressive disorder and negative affective stimuli has recently been shown to be associated with hypoconnectivity between the hippocampus, amygdala, right orbitofrontal cortex, and the hypothalamus (Garcia et al., 2020). In the same subpopulation hyperactivity of the right amygdala and the hypothalamus was observed, suggesting that cardiac autonomic dysregulation is linked to emotional stress in women.

Activation of inflammatory processes affects cardiovascular outcomes in both, stable and unstable angina, irrespective of myocardial damage and conventional risk markers of CVD (de Lemos et al., 2003; Gebhard et al., 2017; Kalkman et al., 2018). Similarly, levels of inflammatory biomarkers have been shown to be sex- and ethnicity-dependent (Khera et al., 2005) with distinct upregulation of inflammatory cells, C-reactive protein (CRP) and interleukin-6 (Thorand et al., 2006). Moreover, the recent CANTOS trial, which assessed the safety and efficacy of the anti-inflammatory drug Canakinumab in patients with previous myocardial infarction, reported a significantly reduced event rate in the test group, as compared to the placebo arm (Ridker et al., 2017). In a secondary analysis from the CANTOS trial, the authors unveiled that women and men both benefited from canakinumab treatment, as shown by the composite cardiovascular endpoint encompassing recurrent non-fatal myocardial infarction, non-fatal stroke, cardiovascular death and unstable angina (Ridker et al., 2018). However, the use of anti-inflammatory therapy is still far from application in cardiovascular clinical routine. This is reflected by the inefficacy of methotrexate to treat stable CAD (Ridker et al., 2019). In addition, colchicine has lately been observed to reduce the risk of ischemic cardiovascular events in patients with recent myocardial infarction (Nidorf et al., 2020). Notably, this risk reduction was non-significant in women, as demonstrated by a subgroup analysis. Further, no sex-disaggregated data on adverse effects of colchicine were reported. This is a major omission given that experimental studies in rats indicate a twofold higher susceptibility to lethal effects of colchicine in females (Wiesenfeld et al., 2007). While cardiovascular pathologies and outcomes have been associated with a higher level of inflammatory activity, it was only recently demonstrated that an upregulated metabolic activity of the vertebral bone marrow, a surrogate marker of inflammation, is a strong and independent predictor of myocardial injury, suggesting that inflammatory biomarkers play a significant role in the development and progression of CAD toward myocardial ischemia and fibrosis (Fiechter et al., 2019a). Most intriguingly, these observations were sex-dependent with a significant association of inflammation and myocardial function and perfusion in women, but not in men.

In summary, a sex-specific mechanism linking cardiovascular pathologies with inflammation is so far lacking. Nonetheless, experimental findings suggest a distinct association between sympathetic hyperactivity, upregulation of cytokine cascades, and pathological myocardial remodeling (Xiao et al., 2018). Potential underlying mechanisms may include the toxicity of NLRP3 inflammasome complexes on myocardial cells, which are triggered by sympathetic hyperactivity (Karakas et al., 2018). Further, an elevated body mass index and other CVD risk factors are tightly associated with higher inflammatory states mainly reflected by higher CRP levels in the female, but not in the male population (Wong et al., 2001; Qasim et al., 2011; Pruijm et al., 2013; Shanahan et al., 2014). Finally, adjustment for CRP leads to a loss of a positive association between atherosclerotic plaque eccentricity and epicardial fat in women. This link further points toward a detrimental association between inflammation and adipose tissue in women (Miao et al., 2011; Haider et al., 2019b). In conclusion, there is an unmet medical need for tailored preventive strategies in women to assess their residual inflammation-related cardiovascular risk (Gencer and Mach, 2019).

## The Neuro-Inflammatory- Vascular Circuit: Evidence for a Sex-Specific Link?

**FIGURE 2:**
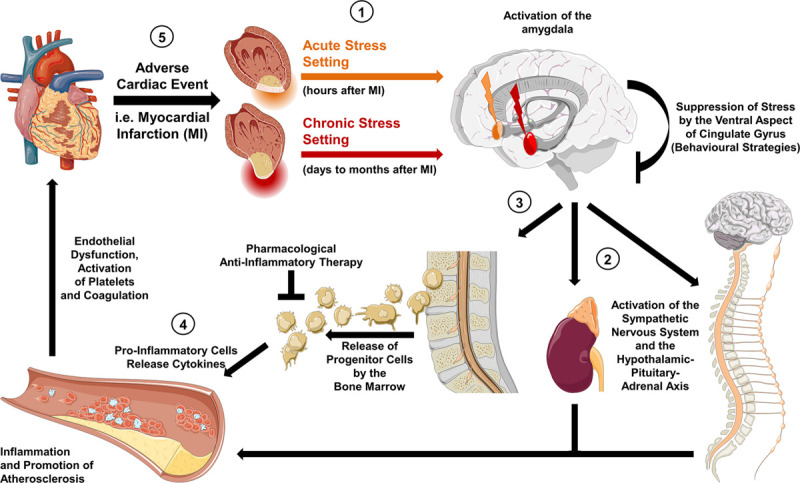
Hypothetic mechanisms of the neuro-inflammatory-vascular circuit. (1) Activity in the brain’s salient network is induced by acute stress (orange arrow) and chronic stress (red arrow), thereby triggering the activation of the amygdala. (2) Efferent projections to the brainstem upregulate sympathetic activity as well as the hypothalamic-pituitary-adrenal axis leading to an increase in circulating norepinephrine and cortisol. In an acute setting this results in an increased heart rate and blood pressure, which can be pharmacologically targeted by sympatholytic agents. In a chronic state, the tonic firing of the amygdala leads to modulation of central brain structures, such as the locus coeruleus, and to a chronic inflammatory state. (3) This is reflected by enhanced bone marrow activity and release of progenitor inflammatory cells. (4) Inflammatory cytokines (interleukin-6 and interleukin-18) promote endothelial dysfunction and progression of atherosclerosis. (5) The latter increases the risk of adverse cardiac events. Targeted anti-inflammatory treatment or behavioral strategies (e.g., stress reduction concepts) have the potential to diminish detrimental effects on the cardiovascular system.

Currently, deleterious sex-specific effects of enhanced psychological stress on cardiovascular health are largely unexplored. However, in an experimental mouse model, Xiao et al. (2018) linked fibrotic myocardial remodeling to myocardial sympathetic hyperactivity, with the latter prompting inflammasome-dependent release of interleukin-18. This observation supports a strong relation between activation of the autonomous nervous system, inflammatory state, and adverse cardiovascular outcomes. Moreover, a recent investigation on the association between central neural structures, inflammation, and myocardial injury revealed for the first time a sex-specific link between enhanced resting amygdalar metabolic activity, upregulated inflammatory state, reflected by heightened bone marrow activity, and myocardial function, i.e., presence of myocardial scar and left ventricular dysfunction (Figure 2; Fiechter et al., 2019b). These findings add to the body of evidence concerning the concept of sexual dimorphism in emotional stress responses, mediated by the limbic system.

A hypothesis with regard to the potential mechanism interconnecting the brain with the heart has recently been suggested by Mehta et al. (2019). The authors proposed a malignant cascade of events following a stress trigger consisting of interconnected areas of the brain such as the prefrontal cortex, the amygdala, the hippocampus, and the locus coeruleus. In detail, psychological stress stimulated the amygdala, which in turn activated the hypothalamus-pituitary-adrenal axis by the sympathetic nervous system and its sites of control, i.e., nucleus tractus solitarius, the parabrachial nucleus, the rostroventral lateral medulla, and the dorsal motor nucleus of the vagal nerve (Vaccarino and Bremner, 2017; Bremner et al., 2018). This activation may then result in increased levels of epinephrine, norepinephrine, and cortisol leading to an activation of the body’s “fight and flight” system such as an upregulated heart rate and blood pressure (Vaccarino and Bremner, 2017). During states of chronic stress exposure, executive functions of the medial prefrontal cortex are tuned down, while amygdalar activity is enhanced and thus, continuous activation of the noradrenergic locus coeruleus was observed (Arnsten et al., 2015). This persistent amygdalar firing may lead to chronic sympathetic hyperactivity, prompting an upregulated inflammatory state as well as subsequent endothelial dysfunction and atherosclerosis (Lima et al., 2019).

## Conclusion

Notwithstanding the substantial progress that has been made in CVD management, current reports still point to a persistent knowledge gap concerning optimized management and risk-stratification in women, particularly with regard to ACS. One of the major challenges to close this gap is to reduce the persistent underrepresentation of females in cardiovascular clinical trials (Scott et al., 2018). Further, the neuro-inflammatory-vascular circuit appears to contribute disproportionally to cardiovascular risk in women and men. Specifically, emotional stress-induced amygdalar activity leading to upregulated inflammatory pathways, negatively affects the heart’s function and perfusion in the female, but not in the male population. This mechanism might further contribute to the development of Takotsubo cardiomyopathy, thereby explaining the predominance of women in Takotsubo cardiomyopathy. Finally, there is a plethora of unanswered questions concerning the impact of socio-environmental and contextual factors on gender-associated manifestation of ACS presentation and outcomes. Thus, further work is needed to identify interrelating molecular pathways of this cascade with the ultimate aim to develop pharmacologic and behavioral therapies that are tailored to women and men.

## Future Perspectives

Despite the fundamental knowledge gaps that currently exist in our understanding of the mechanisms underlying brain-heart interactions in CVD patients, there is a growing interest to dissect the pathways involved. As such, initial efforts have been undertaken to understand the role of enhanced baseline inflammation and sympathetic hyperactivity in patients with adverse cardiovascular outcomes. Although an enhanced amygdalar metabolic activity has been linked to adverse cardiovascular events, interventional studies are required to establish a direct causality. Further, to dissect underlying molecular mechanisms, it is imperative to address pivotal questions including, but not limited to; (1) What are the key components of the immune system that are affected by amygdalar activation? (2) Does amygdalar activation result in impaired cardiac function and/or abnormal cardiac perfusion by triggering sympathetic hyperactivity? (3) Why are women disproportionally affected by emotional stress and what is the role of sex hormones in governing brain-heart interactions? (4) In addition to the prognostic value of enhanced amygdalar metabolic activity, can the “amygdala-related” cardiovascular risk be modulated by therapeutic intervention? Translational molecular imaging has so far played a critical role in elucidating complex interactions of the brain-heart axis. Nonetheless, a multidisciplinary effort will be required to address the abovementioned questions. Further, prospective studies are warranted to translate these intriguing concepts into a genuine advantage for CVD patients.

## Author Contributions

All authors listed have made a substantial, direct and intellectual contribution to the work, and approved it for publication.

## Conflict of Interest

All authors have the following to disclose: The University Hospital of Zurich holds a research contract with GE Healthcare. CG has received research grants from the Novartis Foundation and speaker’s fees from Sanofi Genzyme, Switzerland.
